# Clinical presentation, diagnosis, and treatment of chronic granulomatous disease

**DOI:** 10.3389/fped.2024.1384550

**Published:** 2024-06-28

**Authors:** Olga Staudacher, Horst von Bernuth

**Affiliations:** ^1^ Department of Pediatric Respiratory Medicine, Immunology and Critical Care Medicine, Charité—Universitätsmedizin Berlin, Humboldt-Universität zu Berlin, and Berlin Institute of Health, Berlin, Germany; ^2^ Department of Immunology, Labor Berlin—Charité Vivantes, Berlin, Germany; ^3^ Berlin Institute of Health (BIH), Charité—Universitätsmedizin Berlin, Berlin, Germany; ^4^ Berlin-Brandenburg Center for Regenerative Therapies (BCRT), Charité—Universitätsmedizin Berlin, Humboldt-Universität zu Berlin, and Berlin Institute of Health (BIH), Berlin, Germany

**Keywords:** chronic granolumatous disease, diagnosis, clinical presentation, HSCT, hematopoietic stem cell transplantation, therapy

## Abstract

Chronic granulomatous disease (CGD) is caused by an impaired respiratory burst reaction in phagocytes. CGD is an X-linked (XL) (caused by pathogenic variants in *CYBB*) or autosomal recessive inborn error of immunity (caused by pathogenic variants in *CYBA*, *NCF1*, *NCF2*, or *CYBC1*). Female carriers of XL-CGD and unfavorable lyonization may present with the partial or full picture of CGD. Patients with CGD are at increased risk for invasive bacterial and fungal infections of potentially any organ, but especially the lymph nodes, liver, and lungs. Pathogens most frequently isolated are *S. aureus* and *Aspergillus* spp. Autoinflammation is difficult to control with immunosuppression, and patients frequently remain dependent on steroids. To diagnose CGD, reactive oxygen intermediates (O_2_^−^ or H_2_O_2_) generated by the NADPH oxidase in peripheral blood phagocytes are measured upon *in vitro* activation with either phorbol-12-myristate-13-acetate (PMA) and/or TLR4 ligands (*E. coli* or LPS). Conservative treatment requires strict hygienic conduct and adherence to antibiotic prophylaxis against bacteria and fungi, comprising cotrimoxazole and triazoles. The prognosis of patients treated conservatively is impaired: for the majority of patients, recurrent and/or persistent infections, autoinflammation, and failure to thrive remain lifelong challenges. In contrast, cellular therapies (allogeneic stem cell transplantation or gene therapy) can cure CGD. Optimal outcomes in cellular therapies are observed in individuals without ongoing infections or inflammation. Yet cellular therapies are the only curative option for patients with persistent fungal infections or autoinflammation.

## Historical perspectives

The first clinical accounts of CGD date back to 1954 and 1957, when children with increased susceptibility to bacterial infections in the lungs, lymph nodes, and skin and granulomatous lesions were described. These patients showed pigmented lipid histiocytes in granulomas of visceral tissues and hypergammaglobulinemia in peripheral blood. The disease was named “fatal granulomatous disease of childhood,” later changed to its current name, “chronic granulomatous disease” (CGD) (1–3). Antibiotic treatment and surgical drainage of abscesses improved life expectancy from less than 10 years to less than 20 years. Until the late 1960s, the presence of chronic granulomas with typical lipid-laden histiocytes remained the only way to diagnose CGD. In 1967 Robert Baehner and David Nathan described how “intact leucocytes of two children with chronic granulomatous disease fail to reduce nitroblue tetrazolium (NBT) during phagocytosis.” They also established this failure of phagocytes to reduce NBT as the first screening test for CGD (4, 5). At the same time, clinical observations discovered CGD not only in boys but also in girls, suggesting X-linked as well as autosomal recessive inheritance for CGD (6, 7). In the 1970s it became evident that phagocytes from patients with CGD lack a functional NADPH oxidase and that these phagocytes cannot form superoxide upon activation (8–10). Aspergillosis, BCGitis upon vaccination with BCG, and non-infectious (auto)inflammatory manifestations of CGD were clinically relevant issues that were first addressed 20 years after the initial descriptions of mainly bacterial infections (11, 12). Life expectancy improved further upon the introduction of antibiotic prophylaxis directed against bacteria with cotrimoxazole (13), and against aspergillosis with itraconazole (14, 15). Early observations indicated that CGD can be cured through allogeneic bone marrow transplantation. However, initial results were not considered sufficiently encouraging to offer this option upfront to every individual with CGD (16–18).

## Molecular genetics of chronic granulomatous disease

**Figure 1:**
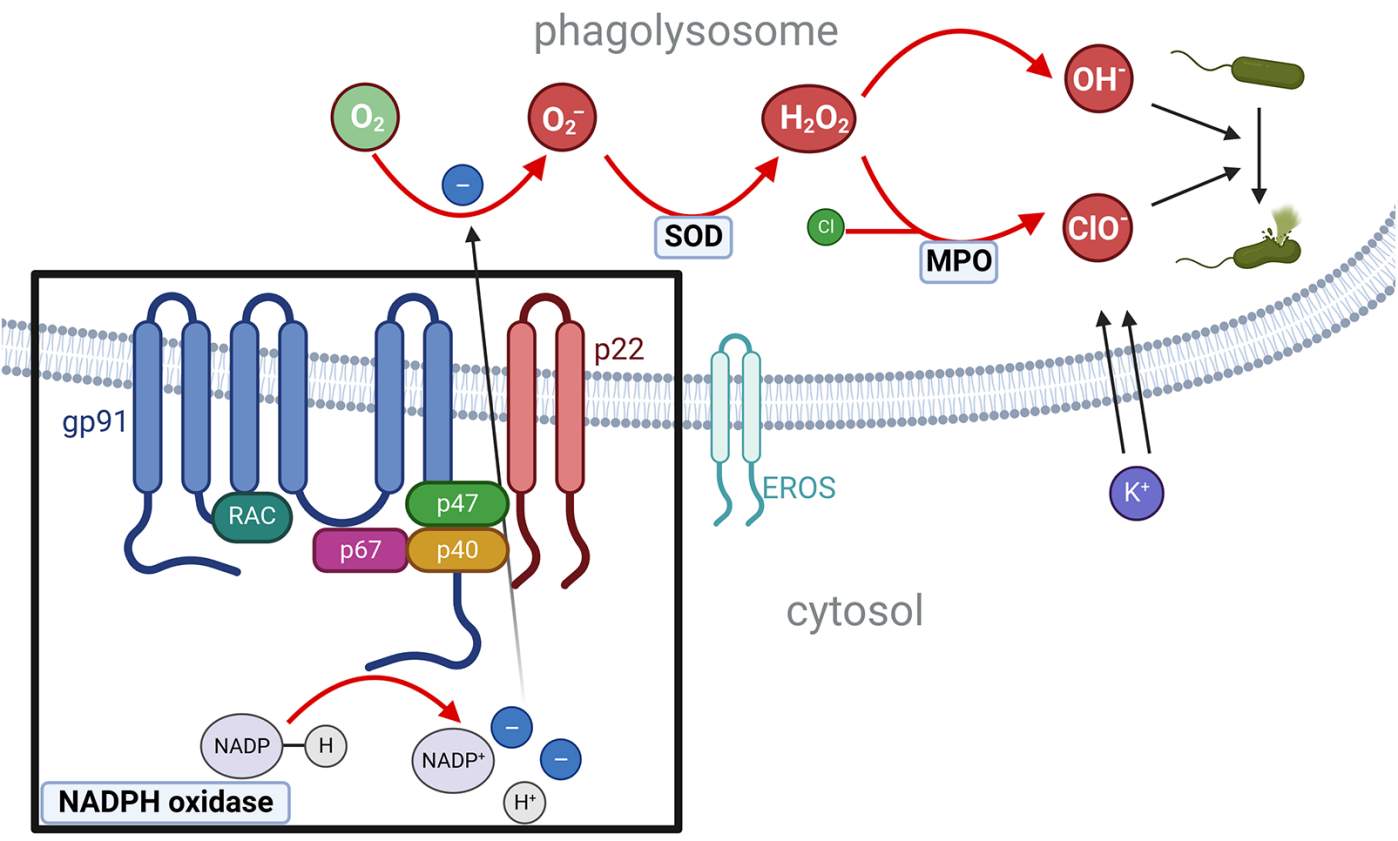
NADPH oxidase complex and the production of ROS.

Pathogenic variants in genes encoding subunits or regulatory proteins of the phagosomal NADPH oxidase complex cause CGD. The NADPH oxidase core complex consists of six proteins: the membrane-bound catalytic subunit gp91^phox^ (coded by *CYBB*), the membrane-bound activator p22^phox^ (coded by *CYBA*), the regulatory subunits p47^phox^ (coded by *NFC1*), p67^phox^ (coded by *NCF2*), p40^phox^ (coded by *NCF4*), and the GTPase RAC (in 96% of cases RAC2, RAC1 is also possible) (19). EROS (coded by *CYBC1*) is a chaperone protein for the dimerization of gp91^phox^ and p22^phox^ (20, 21). Upon activation, the phagosomal NADPH oxidase catalyzes the reaction of NADPH to form a cationic NADP^+^, a proton (H^+^), and two electrons. The electrons then enter the phagolysosome. There, oxygen molecules are reduced to two superoxide anions (O_2_^−^) (NADPH + 2O_2_⇔ NADP^+^ + 2O_2_^−^ + H^+^). A subsequent reaction catalyzed by a superoxide dismutase (SOD) [or alternatively myeloperoxidase (MPO)] produces superoxide peroxide (H_2_O_2_). The superoxide peroxide then either spontaneously decomposes into two superoxide ions (OH^−^) or, in a second reaction catalyzed by MPO, combines with a chloride molecule to form hypochlorite (ClO^−^). Both reactive oxygen species (ROS) not only lyse bacteria but also cause a pH-dependent influx of K^+^, which subsequently activates proteases, crucial for the destruction of bacteria (Figure 1) (22). X-linked pathogenic variants in *CYBB* or autosomal recessive pathogenic variants in *CYBA, NCF1, NCF2*, and *CYBC1* lead to absent or severely reduced production of superoxide in all phagocytes (neutrophils as well as monocytes, macrophages, and eosinophils) (23–25).

Due to a large deletion in the X chromosome, patients with the X-linked form of the disease may display a contiguous gene defect causing the McLeod phenotype in erythrocytes, as well as Duchenne muscular dystrophy or retinitis pigmentosa. Patients with the McLeod phenotype must not receive repeated transfusions with erythrocytes expressing K20 and XK1. Acanthocytosis of erythrocytes in the peripheral blood may be indicative of the McLeod phenotype, but its absence does not rule it out (26–28).

ROS serve immunoregulatory functions in addition to their antimicrobial effect. The activation of ataxia-telangiectasia-mutated (ATM) kinase, for instance, requires NADPH oxidase (29). NADPH-deficient phagocytes show autophagic dysfunction, increased production of IL-1β upon activation with LPS, and an altered interferon signature. CGD patients show impaired apoptosis of peripheral blood neutrophils, yet not of monocytes (30–33).

P40^phox^ deficiency, caused by autosomal recessive pathogenic variants in *NCF4*, is a similar but distinct disease. Neutrophils with p40^phox^ deficiency exhibit impaired burst activity, while macrophages are less affected and demonstrate a greater capacity for producing reactive oxygen intermediates than in patients with bona fide CGD. As a result, patients with p40^phox^ deficiency exhibit a far milder clinical phenotype. To date, no patients with p40^phox^ deficiency have been described as suffering from invasive bacterial and fungal infections (34–36). Dominant negative mutations in *RAC2* result in a phenotype resembling leukocyte adhesion deficiency with an impaired oxidative burst, as RAC2 is not only crucial for NADPH oxidase but also controls cytoskeleton formation and cell adhesion (37).

## Clinical presentation—infections and autoinflammation in CGD

**Figure 2:**
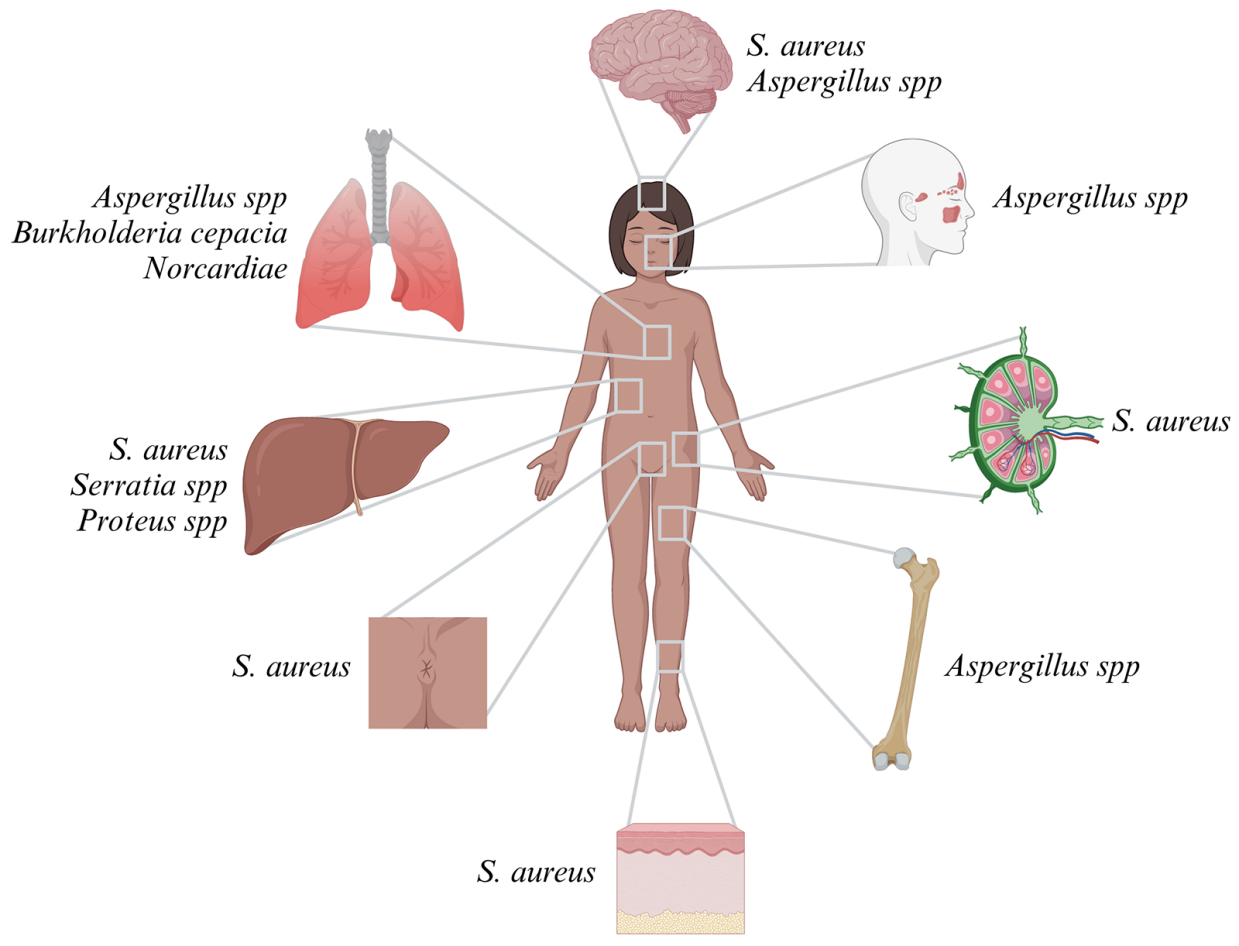
Infection sites and most common pathogens in CGD.

Patients with CGD are at increased risk for invasive bacterial and fungal infections. Symptoms usually start in infancy, with a median age at diagnosis of CGD between 2.5 and 3 years. However, some patients are not identified until adolescence or adulthood (38–40). The infectious phenotype may differ significantly depending on local climate and antimicrobial resistance patterns. The most common pathogens are (from most to least common) *Staphylococcus aureus* (*S. aureus*), *Aspergillus* spp., *Burkholderia cepacia*, *Serratia* spp., *Nocardia*e, and *Salmonella* spp (38, 39, 41–45). Staphylococcal infections mostly affect the skin, lymph nodes, rectum, and brain. *Burkholderia cepacia* manifests in the lungs and can cause the life-threatening Cepacia syndrome, a condition similar to a cytokine storm in macrophage activation syndrome. *Serratia* and *Proteus* spp. cause liver abscess, and infections from *Nocardia* most often affect the lungs (38, 39, 41, 44–46). Molds, such as e.g., *Histoplasma* spp., *Phellinu*s spp., *Rasamsonia* spp., *Rhizopus* spp., and *Trichosporon* spp., and, far less commonly, *Mucormucosis*, pose particular threats (47–52). Invasive mold infections mainly affect the lungs, brain, bones (as osteomyelitis), and nasal cavities (Figure 2) (53, 54). BCGitis is another common presenting symptom in regions where infants are routinely vaccinated with BCG (55, 56). Yet neither mycobacteria other than tuberculosis (MOTT) nor bona fide tuberculosis infections are common in CGD (38, 39, 45, 57, 58).

**Figure 3:**
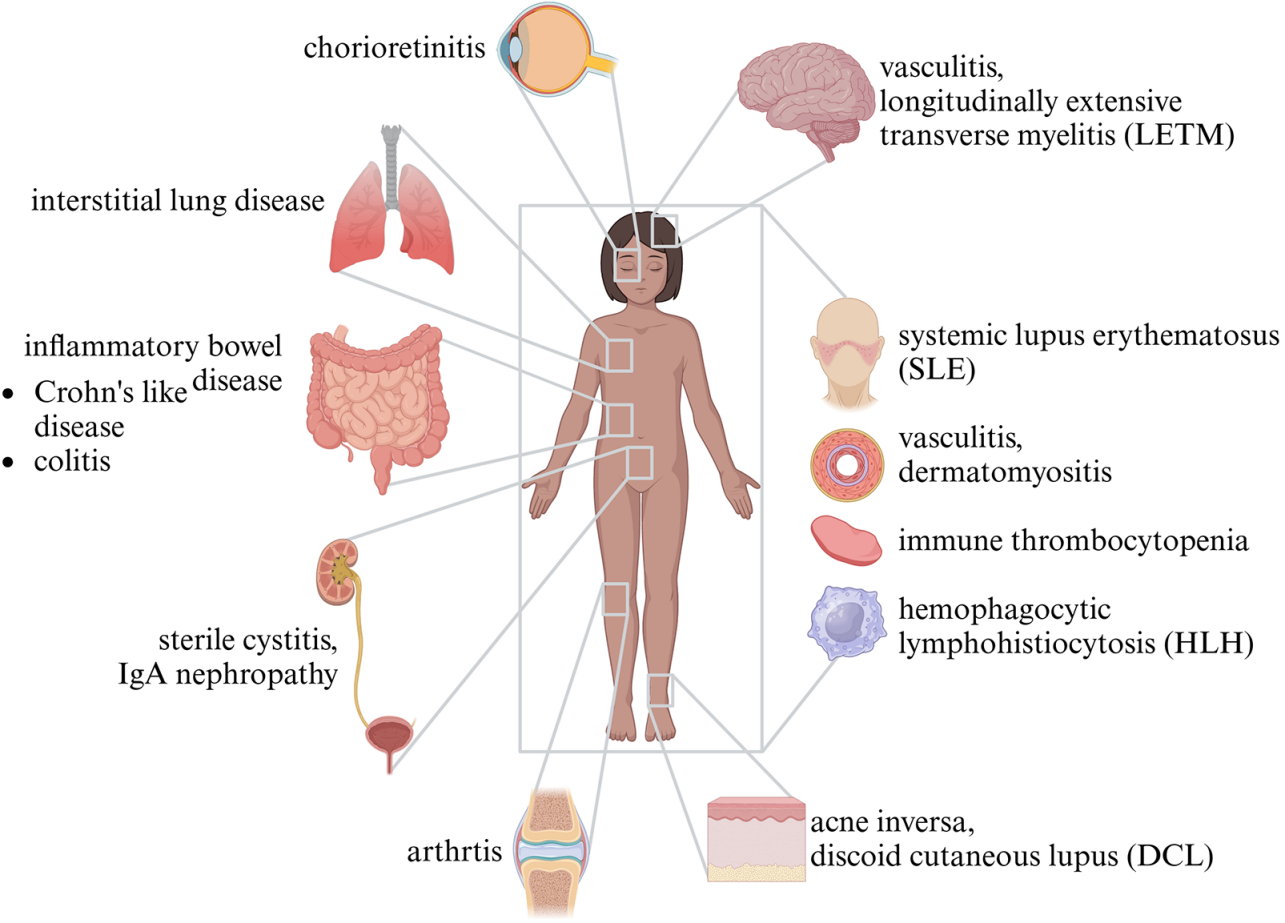
Autoinflammation in CGD.

The eponymous granulomas of CGD are often found in the urinary tract, intestines, or lungs. They can lead to stenosis in hollow organs, or fibrosis. Granuloma formation can be seen as a mechanism by which the immune system contains infections, yet it is noteworthy that many granulomas found in patients with CGD are sterile and of autoinflammatory origin (59–61). About half the patients with CGD not only suffer from infections but also develop autoinflammation or immune dysregulation. Inflammatory bowel disease is the most common manifestation and may resemble Crohn's disease (62). Histology, however, may differ from Crohn's disease by pigmented lipid histiocytes, microgranulomas, and eosinophilic abscesses (63, 64). Further organs potentially affected by autoinflammation are the urinary tract/bladder and the lungs, but also sites like the brain, joints, and retina. Incident patients with CGD and systemic conditions resembling systemic lupus erythematosus, vasculitis, sarcoidosis, and thrombocytopenia were also described (59, 65–68) (Figure 3).

It is important to consider CGD in patients presenting with infections at any of the above sites. But in countries with endemic tuberculosis, the presence of difficult-to-treat or disseminated tuberculosis should prompt an evaluation for CGD (69). As Crohn's-like disease is common in CGD and may develop before the onset of invasive fungal or bacterial infections, CGD must be ruled out in any patient with Crohn's disease (64, 70–72). Similarly, CGD should be ruled out in patients with sterile granuloma formation (73–75).

Important differential diagnoses of CGD are autosomal dominant hyper-IgE syndrome caused by dominant negative mutations in *STAT3* (“DN-STAT3-HIES”) and MyD88/IRAK4 deficiency. DN-STAT3-HIES is often associated with abscesses and pneumonia caused by *S. aureus*. Infections with *Aspergillus* spp. are much less common, and invasive aspergillosis is extremely rare in DN-STAT3-HIES, but aspergillomas may occur in the context of characteristic pneumatoceles, which are not typically seen in CGD (76). In contrast, chronic mucocutaneous candidiasis is common. In addition, DN-STAT3-HIES is associated with skeletal abnormalities and severe dermatitis, both of which are not features of CGD (77–79). On the other hand, patients with MyD88/IRAK4 deficiency often experience invasive infections with *Streptococcus pneumoniae*, *S. aureus*, and *Pseudomonas aeruginosa*, but fungal infections are rare (80–82). In contrast to patients with CGD, patients with DN-STAT3-HIES and MyD88/IRAK4 deficiency have impaired IL-6 signaling or production. This leads to cold abscesses in DN-STAT3-HIES and to low CrP despite invasive infections in MyD88/IRAK4 deficiency (77, 82–85).

## Diagnostic workup and pitfalls

**Figure 4:**
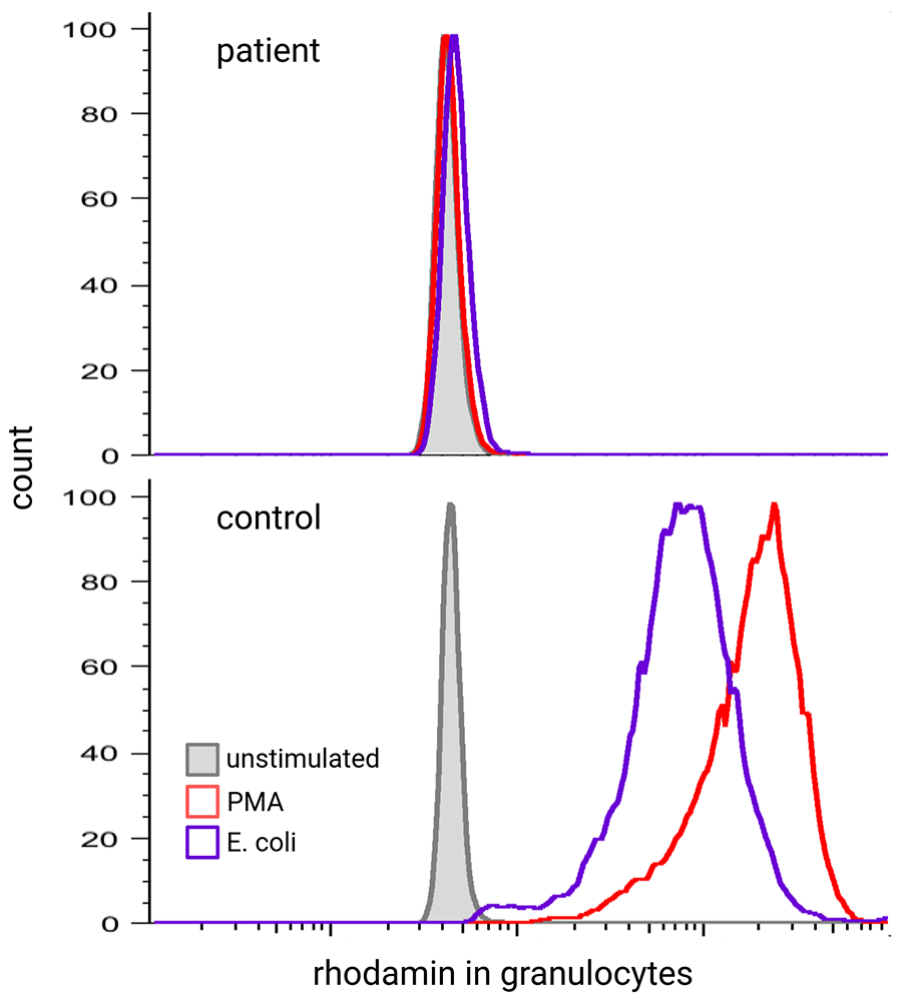
Respiratory burst of a healthy control and patient with CGD measured by DHR assay.

To diagnose CGD, ROS (O_2_^−^ or H_2_O_2_) generated by the NADPH oxidase in phagocytes of peripheral blood are measured upon *in vitro* activation with either phorbol-12-myristate-13-acetate (PMA) and/ or TLR4-ligands (*E. coli* or LPS). We suggest that the assay be repeated at least once and that two different stimuli for the induction of the respiratory burst be used. ROS can be detected by different assays. O_2_^−^ can be measured by chemiluminescence or the seminal NBT assay and H_2_O_2_ by the FACS-based DiHydroRhodamine (DHR) assay (Figure 4) (86, 87). While it was once considered the gold standard for the diagnosis of CGD, NBT has since been surpassed by the flow-cytometry-based DHR assay in terms of time effectiveness, sensitivity, and quantification. However, the NBT assay remains a cost-effective and relatively easy-to-perform option, requiring only a microscope, a stimulant, and NBT. Especially in low-resource settings, the NBT assay is still a highly effective diagnostic tool (88–90).

Functional assays that measure the respiratory burst in phagocytes may be false positive in patients treated with paracetamol/acetaminophen, metamizole, or mesalazine (5-ASA) (91–93). Furthermore, CGD must be distinguished from MPO deficiency, which is not considered an inborn error of immunity. In CGD, the oxygen burst reaction is impaired in all phagocytes, whereas in MPO deficiency, the oxygen burst reaction is only impaired in neutrophils (94).

In patients with a repeatedly impaired respiratory burst, at least the seven genes *CYBA*, *CYBB, NCF1*, *NCF2*, *NCF4 CYBC1*, and *RAC2* should be analyzed for causative mutations.

## Conservative vs. curative treatment of CGD

Upon diagnosis, all patients with CGD should receive conservative treatment with antibacterial and antifungal prophylaxis with cotrimoxazole and triazoles. Cotrimoxazole significantly reduces the incidence of bacterial infections while being relatively inexpensive (95). Itraconazole is still the most widely used agent to prevent invasive *Aspergillus* and other mold infections, but posaconazole may be a choice to consider depending on local resistance patterns (15). Female carriers with unfavorable lyonization of pathogenic variants in the X-linked *CYBB* may develop partial or complete clinical manifestation of CGD (38, 96–98). Given the documented occurrence of serious bacterial infections in patients with ROS below 10%, we propose considering prophylactic treatment against bacteria when ROS levels are below 20% and against molds when ROS levels are below 5% (38, 96, 98). We recommend regularly assessing the respiratory burst of carriers, with a suggested interval of 5 years (96).

Especially in North America, but less so in Europe, a significant number of patients are treated with IFN-γ (99). It has been shown to improve the splicing efficiency of *CYBB* (100). A recent meta-analysis showed a significant reduction in the likelihood of infection, but to date there is insufficient evidence of clinical improvement in patients with CGD on IFN-γ and data on long-term effects are lacking (101). The question of whether there is a general benefit of IFN-γ for all patients with CGD remains unanswered. Treatment is costly and not without side effects (mainly fever, but also mental impairment). In our opinion, IFN-γ can be considered in patients with particular pathogenic variants of *CYBB*.

Hygienic conduct includes avoiding exposure to molds. Patients with CGD should therefore abstain from doing agricultural work and gardening (including composting, mucking out stables, working in barns, etc.) as well as demolition of moist walls. Alternatively, patients must wear an FFP3 mask to prevent mold from entering their airways (102). We do not advise people with CGD to keep pets, although the risk may be manageable if there is strict adherence to hygiene (103). Furthermore, patients undergoing conservative treatment derive substantial benefits from regular follow-up by physicians experienced in CGD care (102). In our European setting, we strive for intervals of 3 months between visits. Although anti-infective prophylaxis greatly reduces mortality, infections still occur at a rate of 0.26–0.64 per patient-year with a cumulative lifetime risk of 20%–40% for aspergillosis, which remains the leading cause of death (39, 43).

Immunosuppressive therapy can become necessary to control autoinflammation and immune dysregulation (59, 104). For mild IBD in CGD, sulfasalazine or alternative aminosalicylates are commonly used as initial treatments (105). Recently, monoclonal antibodies targeting pro-inflammatory cytokines (such as infliximab, anakinra, adalimumab, and ustekinumab) have been explored (106–109). However, the available evidence is limited and ustekinumab is the only treatment that has shown somewhat favorable outcomes (109). Despite their known adverse effects, patients often remain dependent on corticosteroids to control IBD.

In retrospective studies, the median life expectancy of conservatively treated CGD patients is between 30 and 40 years. Additionally, quality of life and academic and professional achievements are severely impaired on conservative treatment (39, 110, 111). In contrast, allogenic hematopoietic stem cell transplantation (HSCT) can potentially cure CGD. Because transplantation-related mortality was originally at 15%–50%, HSCT was historically considered a salvage therapy only for patients with recurrent infections or refractory inflammation (112, 113). Improved HLA matching, fludarabine-based reduced-toxicity conditioning, and the accumulation of clinical experience in guiding patients with CGD through allogenic HSCT have reduced treatment-related morbidity and mortality (Table 1) (115–121). Over the past decade, transplant series have reported survival rates of 83%–96% in CGD patients following matched donor transplantation (115, 116). Several studies have compared the prognosis of CGD patients treated conservatively with those treated with HSCT. A Swedish study of 41 patients reported a superior outcome with HSCT (93% vs. 74% survival) (114). Other studies, including our own retrospective European study, still failed to describe a clearly better survival after HSCT, with survival rates ranging from 76%–90% in both cohorts (41, 43, 45, 115, 118). However, significant reductions in infectious episodes and catch-up growth after HSCT are clearly evident in data from the UK, US, and Europe (43, 45, 115). In particular, patients between the ages of 5 and 14 without active complications at the time of HSCT have excellent outcomes (45, 115, 119, 120). Therefore, all young CGD patients with a ≥9/10 HLA-matched available donor should be considered for HSCT early in life, before chronic sequelae caused by infections and/or autoinflammation occur. Patients for whom no ≥9/10 HLA-matched donor is available can be offered haploidentical HSCT from an unaffected parent [published experience reviewed in (122)]. The idea of early transplantation also holds true for symptomatic X-linked carriers of mutations in CYBB and an unfavorable lyonization (96, 121). HSCT is also possible in older patients after recurrent or persistent infection or autoinflammation and may be the only life-saving option (96, 116, 117).

**Table 1:** Survival rates in CGD patients treated with HSCT vs. patients on conservative treatment (non-HSCT).

Author (year)	Survival (%)		Patients (*n*)
	HSCT	Non-HSCT	HSCT/ non-HSCT
van der Berg et al. (2009) (41)	81	74	24/307
Åhlin et al. (2013) (114)	93	46	14/27
Cole et al. (2013) (43)	90	90	30/32
Yonkof et al. (2019) (115)	88	85	50/457
Dedieu et al. (2021) (45)	88	87	50/54
Horwitz et al. (2001) (112)	70	–	10/−
Seger et al. (2002) (113)	85	–	27/−
Güngör et al. (2014) (116)	93	–	56/−
Morillo-Gutierrez et al. (2016) (117)	91	–	70/−
Parta et al. (2017) (118)	82.5	–	40/−
Lum et al. (2019) (119)	89	–	55/−
Chiesa et al. (2020) (120)	86	–	712/−
Tsilifis et al. (2023) (121)	71	–	7/−

Gene therapy for CGD remains limited to trials in only a few highly specialized centers around the world. It is often mentioned as a future alternative cellular therapy “for patients without a suitable donor” (123, 124). But at least most children can be treated by HSCT from a haploidentical parent (122, 125–127). Gene therapy is also promoted for patients with a “high disease burden,” as conditioning is milder than with HSCT (123). However, in the most recent trials, the mortality rates for gene therapy are still higher than those for HSCT. This is presumably due to the disease burden of the patients enrolled, but it may also be because of a high rate of graft rejection in gene therapy (120, 124, 128). To date, there is a lack of long-term data and head-to-head trials, or at least meta-analyses, comparing gene therapy with haploidentical HSCT in patients for whom a matched donor is not available (129). Nevertheless, gene therapy may emerge as an alternative to HSCT in selected situations (124, 130).

## Summary

Chronic granulomatous disease (CGD) is caused by an impaired respiratory burst reaction of all phagocytes rather than an impaired burst in neutrophilic granulocytes only. CGD is an X-linked (caused by pathogenic variants in *CYBB*) or autosomal recessive inborn error of immunity (caused by pathogenic variants in *CYBA*, *NCF1*, *NCF2*, or *CYBC1*). Patients with CGD are at increased risk for bacterial and/or fungal invasive infections of any organ, but mainly the lymph nodes, liver, and lungs. The leading pathogens isolated are *S. aureus* and *Aspergillus* spp. But *Serratia*, *Proteus* spp., *Burkholderia cepacia*, *Nocardia* spp., and *Salmonella* spp. are still often isolated, and infections with almost any intracellular bacteria and fungi are possible. Infections with *Aspergillus* spp. and *Burkholderia cepacia* remain the major cause of morbidity and mortality in patients on conservative treatment. Patients often develop skin infections by *S. aureus*. Autoinflammation, and inflammatory bowel disease in particular, is difficult to control by immunosuppression, and patients frequently remain dependent on steroids. Female carriers of pathogenic variants in *CYBB* and unfavorable lyonization may present with the partial or even full picture of CGD. For the diagnosis of CGD, reactive oxygen intermediates (O_2_^−^ or H_2_O_2_) generated by the NADPH oxidase in phagocytes of peripheral blood are measured upon *in vitro* activation with either PMA and/ or TLR4 ligands (*E. coli* or LPS). These assays may be false positives in patients treated with paracetamol, metamizol or mesalazine (5-ASA). Conservative treatment must adhere to a strict hygienic conduct and antibiotic prophylaxis against bacteria and fungi. Cotrimoxazole and triazoles that work against intracellular bacteria and *Aspergillus* spp. are the mainstay of the latter. With ongoing advancements in diagnostics, prophylaxis, and therapeutic modalities, it is plausible that life expectancy may surpass the age range of 30–40 years in conservatively treated patients. Most patients with CGD who receive conservative treatment, however, face lifelong challenges such as recurrent and/or persistent infections as well as steroid-dependent autoinflammation and subsequently failure to thrive. Overall, this leads to an unfavorable psychosocial prognosis. In contrast, cellular therapies (allogenic HSCT from a healthy donor or autologous gene therapy-modified cells) can cure CGD. HSCT in individuals without ongoing infections or inflammation offers a fair, yet unfortunately still far from completely event-free prognosis and chance for overall survival. But neither persistent infections nor refractory autoinflammation are a contraindication against HSCT; rather, they are an indication to proceed to a definite cure through cellular therapy. If no HLA-matched donor is available, most infants and children can be transplanted from a haploidentical parent. If no such donor is available, gene therapy may be an alternative option.
